# Biological and Genetic Heterogeneity in *Trypanosoma dionisii* Isolates from Hematophagous and Insectivorous Bats

**DOI:** 10.3390/pathogens9090736

**Published:** 2020-09-07

**Authors:** Juliana Helena da Silva Barros, André Luiz Rodrigues Roque, Samanta Cristina das Chagas Xavier, Kátia Cristina Silva Nascimento, Helena Keiko Toma, Maria de Fatima Madeira

**Affiliations:** 1 Laboratório de Biologia de Tripanosomatídeos, Instituto Oswaldo Cruz, Fundação Oswaldo Cruz, Rio de Janeiro 21040-360, Brazil; roque@ioc.fiocruz.br (A.L.R.R.); samanta@ioc.fiocruz.br (S.C.d.C.X.); katiacristh@gmail.com (K.C.S.N.); 2 Laboratório de Diagnóstico Molecular e Hematologia, Faculdade de Farmácia, Universidade Federal do Rio de Janeiro, Rio de Janeiro 21491-599, Brazil; hktoma@globo.com; 3 Laboratório de Vigilância em Leishmanioses, Instituto Nacional de Infectologia Evandro Chagas, Fundação Oswaldo Cruz, Rio de Janeiro 21040-360, Brazil; fatima.madeira2@gmail.com

**Keywords:** Chiroptera, trypanosomatid, *Schizotrypanum*, integrative methodologies, molecular characterization, biological characterization, differences among isolates

## Abstract

This study describes the morphological, biochemical, and molecular differences among *Trypanosoma dionisii* isolates from hemocultures of hematophagous (*Desmodus rotundus*; *n* = 2) and insectivorous (*Lonchorhina aurita*; *n* = 1) bats from the Atlantic Rainforest of Rio de Janeiro, Brazil. Fusiform epimastigotes from the hematophagous isolates were elongated, whereas those of the insectivorous isolate were stumpy, reflected in statistically evident differences in the cell body and flagellum lengths. In the hemocultures, a higher percentage of trypomastigote forms (60%) was observed in the hematophagous bat isolates than that in the isolate from the insectivorous bat (4%), which demonstrated globular morphology. Three molecular DNA regions were analyzed: V7V8 (18S rDNA), glycosomal glyceraldehyde 3-phosphate dehydrogenase gene, and mitochondrial cytochrome *b* gene. The samples were also subjected to multilocus enzyme electrophoresis and random amplified polymorphic DNA analysis. All isolates were identified as *T. dionisii* by phylogenetic analysis. These sequences were clustered into two separate subgroups with high bootstrap values according to the feeding habits of the bats from which the parasites were isolated. However, other *T. dionisii* samples from bats with different feeding habits were found in the same branch. These results support the separation of the three isolates into two subgroups, demonstrating that different subpopulations of *T. dionisii* circulate among bats.

## 1. Introduction

Trypanosomatids are widely distributed in nature and according to the number of hosts participating in their biological cycle, are classified as monoxenous (one host) or dixenous (two or more hosts) [1]. Due to its wide morphological diversity, its taxonomic classification has always been a challenge, since traditional morphological and biological studies, to the current integration with genomics analysis and improvements in microscopy approaches [2]. Among the different genera of the Trypanosomatidae family, the *Trypanosoma* genus includes species that are found in all classes of vertebrate hosts. The protozoan *Trypanosoma dionisii* is a parasite found in the Americas (Brazil, Bolivia, and the United States), Europe (Belgium, England, and Czech Republic), and Asia (China and Japan) [3,4,5,6,7,8,9]. In Brazil, *T. dionisii* is found in almost all biomes, including the Amazon, Atlantic Forest, Cerrado, Pantanal, and transition areas of the Pantanal and Cerrado. The protozoan infects different bat families, including Phyllostomidae, Molossidae, Noctilionidae, and Vespertilionidae in various regions from northern to southern Brazil [4,10,11,12,13].

For many decades, *T. dionisii* was considered restricted to bats and believed to be transmitted only by Cimicidae, which acts as a vector [14,15,16]. Recent studies have shown that *T. dionisii* is not restricted to only bats; it also infects different mammalian hosts and vectors, including human cardiac tissue [17], Carnivora clot blood [18], the Didelphimorphia [18] species, and the digestive tract of the triatomine, *Triatoma vitticeps* [17]. These recent descriptions demonstrate that very little information is available about the dynamics of the transmission cycle of this parasite in nature, and the hosts and vectors involved.

*T. dionisii* is categorized into the *Trypanosoma cruzi* clade with other species of the subgenus *Schizotrypanum*, such as *Trypanosoma cruzi cruzi*, *Trypanosoma cruzi marinkellei*, and *Trypanosoma erneyi*. In fact, *T. dionisii* was referred to as *T. cruzi*-like for many decades due to its morphological similarity in terms of blood and cultured forms to *T. c. cruzi* [14]. Both *T. dionisii* and *T. c. cruzi* can invade mammalian cells [19] and alternate developmental forms between hosts, with epimastigotes and metacyclic trypomastigotes appearing in the invertebrate host and bloodstream trypomastigotes and amastigotes found in the mammalian host during the life cycle [16,19,20,21].

Studies evaluating the biochemical and biological characteristics of *T. dionisii* have been undertaken for many decades [22,23,24], and current research focuses on identification and phylogenetic analysis. A number of genetic markers, such as ribosomal RNA gene (rDNA) regions (18S and internal transcribed spacer (ITS)), glycosomal glyceraldehyde 3-phosphate dehydrogenase gene (*gGAPDH*), and mitochondrial cytochrome *b* gene (*Cytb*), can be used for genetic analyses of *T. dionisii* [4,5,25,26,27]. Previous studies have focused on recognizing *T. dionisii* genotypes solely on the basis of molecular data [28]. In this study, integrated analysis of morphological and biochemical (as complementary elements) characteristics, along with molecular data, played an important role in the characterization of *Trypanosoma* species, especially to identify intraspecific differences. These approaches were employed to compare *T. dionisii* isolates obtained from hemocultures: M1014 and M1015 isolates from hematophagous (*Desmodus rotundus*) and M1011 isolate from insectivorous (*Lonchorhina aurita*) bats captured in Rio de Janeiro, Brazil.

## 2. Results

### 2.1. Parasite Morphology and Induction of Metacyclogenesis

Parasites were maintained by weekly passages in Novy, McNeal, Nicolle (NNN)/Schneider´s medium and demonstrated extensive polymorphism among the isolates. Isolates M1014 and M1015 were characterized by a large percentage of trypomastigote forms (60%) in all three intervals of observation (three, 10 and 17 days). After three and 10 days, the presence of elongated epimastigote forms and absence of rosettes could be observed in the culture. After 17 days, many forms were observed rounded and destroyed. The isolate M1011 presented some different characteristics: after three days, it presented globular (rounded) epimastigote forms and an absence of rosettes; after 10 and 17 days, the majority of forms were rounded and dividing. M1011 was also observed in degenerate forms, with an absence of rosettes, and rare trypomastigote forms (4%). After metacyclogenesis and induction in Roswell Park Memorial Institute (RPMI) medium, the percentage of trypomastigote forms in the culture moderately increased (13%).

**Figure 1:**
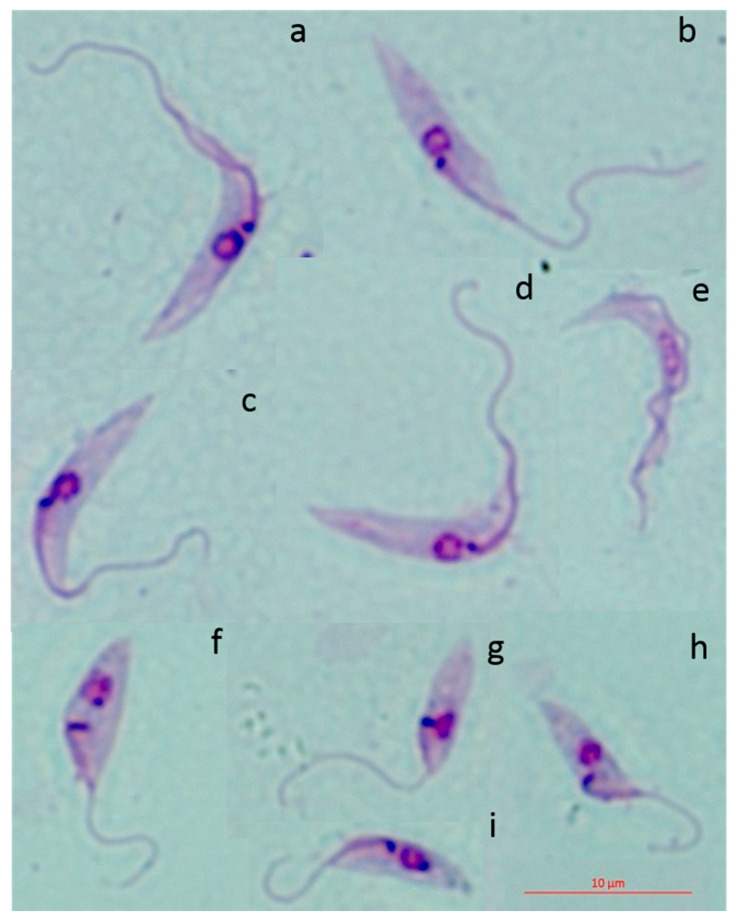
Photomicrographs showing the morphological diversity of cultured forms of *T dionisii* from hematophagous bat M1014 (**a**–**e**) and insectivorous bat M1011 (**f**–**i**). Epimastigote forms (**a**–**d**,**f**–**i**) and trypomastigote form (**e**).

Epimastigote forms obtained from insectivorous bats (M1011) and cultured for 10 days varied in terms of the size of the parasite body, with an average length of 9.29 ± 1.89 µm (6.31–12.71 µm) and size of the free flagellum, with an average length of 9.39 ± 2.19 µm (6.49–12.60 µm). Epimastigote forms from the hematophagous bat (M1014) demonstrated that the parasite body measured 12.97 ± 2.50 µm (6.88–17.76 µm), and the free flagellum measured 11.99 ± 2.50 µm (7.66–17.17 µm; Figure 1). All quantifications of the size of the parasite body and flagellum were in Supplementary Graphic S1. An ANOVA revealed that there were significant differences between body measurement values (*p* = 0.00000297) and flagellum measurements (*p* = 0.0027) between samples M1011 and M1014.

### 2.2. Multilocus Enzyme Electrophoresis

The banding patterns of the *T. dionisii* isolates differed in the glucose phosphate isomerase (GPI), malate dehydrogenase (MDH), isocitrate dehydrogenase (IDH), malic enzyme (ME) isoenzymes, with a clear division into two groups: group 1 consisted of the isolate from *L. aurita* (M1011) and group 2 of the isolates from *D. rotundus* (M1015 and M1014; Supplementary Figure S1). The other five enzymes analyzed showed no differences among the *T. dionisii* isolates.

### 2.3. Molecular Characterization and Sequencing Analysis

Sequence analysis of DNA products obtained with the three molecular targets in comparison with DNA sequences of *Trypanosoma* spp. deposited in Genbank confirmed the identification of the three isolates as *T. dionisii* (Supplementary Table S1).

**Figure 2:**
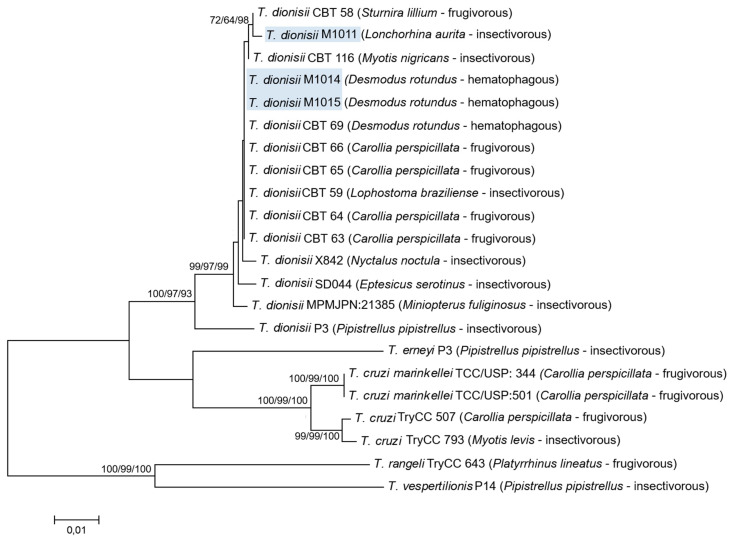
Concatenated phylogenetic tree from combined 18S rDNA and *gGAPDH* data sets (1153 characters) from 12 reference sequences of *Trypanosoma dionisii* and other sequences from the genus *Trypanosoma*. The tree was inferred by using the neighbor-joining (NJ), maximum likelihood (ML), methods and Bayesian inference (BI) based on the Kimura 2-parameter model. Bootstrap test results are shown next to the branches (NJ/ML/BI). Outgroup: *Trypanosoma rangeli and Trypanosoma vespertilionis.* GenBank accession numbers are shown in Table 1.

Phylogenic analysis based on the three different targets demonstrated that the evaluated isolates clustered (100% bootstrap) in a monophyletic assemblage with *T. dionisii* species, generating very similar phylogenetic trees. In the *T. dionisii* clade, the three sequences included in this study were clustered into two separated branches with high bootstraps according to the food habits of the bats in which the parasites were isolated; one branch with the isolate from the insectivorous bat (M1011) and the other with the two isolates from the hematophagous bats (M1014 and M1015). Phylogenetic analysis using a combined dataset of V7V8 18S rDNA and *gGAPDH* (Figure 2) generated similar phylogenetic trees compared to the tree constructed using *Cytb* sequences (Supplementary Figure S2).

Estimates of evolutionary divergence of V7V8 18S rDNA, *gGAPDH*, and *Cytb* nucleotide sequences revealed that *T. dionisii* from the hematophagous bats (M1014 and M1015) were genetically similar. Comparing 18S rDNA, *gGAPDH*, and *Cytb* sequence divergence among the three isolates M1011, M1014, and M1015, the genetic distances obtained were 0.4%, 1.1%, and 6% for 18S rDNA, *gGAPDH*, and *Cytb* sequences, respectively.

### 2.4. Random Amplified Polymorphic DNA (RAPD) 

The dendrogram shows that the three isolates grouped together in a branch separated from the reference samples, but once more, this clade presented two clear branches: one containing isolates from *D. rotundus* (M1014 and M1015) and the other the isolate from *L. aurita* (M1011), supporting the existence of two subgroups (Supplementary Figure S3).

## 3. Discussion

The interest in identifying differences among isolates of the same species of trypanosome began with the discovery of *T. c. cruzi* and its life cycle by Carlos Chagas [29]. To date, most of these studies have concentrated on the *T. c. cruzi* and *T. rangeli* species, as high morphological, biological, biochemical, and molecular variability has been observed in these species [30,31]. Studies regarding differences among *T. dionisii* isolates are scarce, and most recent publications have focused solely on the molecular identification of this parasite in both hosts and vectors from Brazil and some other countries [7,8,10,11,12,18]. The three isolates of *T. dionisii* investigated (M1011, M1014, and M1015) were obtained in a study in different municipalities of Rio de Janeiro, where 22 samples of trypanosomatids were isolated by hemoculture of 84 bats [32]. Three samples were characterized as *T. dionisii* and 19 as *T. madeirae* [33]. Interestingly, we observed that *T. dionisii* and *T. madeirae* [33] were present in the same colony and were infecting the same bat species, *Desmodus rotundus* (data not shown). However, although co-infection is common among bats in nature, it was not detected in this study, probably because the isolation and amplification methods used for the culture of parasites exert a selective force, owing to which mixed infections are not frequently detected.

The classic identification of trypanosomatid protozoans depends on their isolation and subsequent in vitro maintenance; although it is a critical process, it enables morphological, biological, biochemical, and molecular analysis. In this study, the isolation in culture medium allowed the characterization of the three samples as *T. dionisii* using a DNA barcoding approach with three different molecular markers: V7V8 18S rDNA [34], *gGAPDH* [35], and *Cytb* [36]. To the best of our knowledge, this is the first report of *T. dionisii* infecting *Lonchorhina aurita*, an insectivorous bat. 

The integrative methodologies used in this study were in agreement regarding of the overall result that the isolates from *D. rotundus* were similar to each other and different to the isolate from *L. aurita* and support the clear separation of the three isolates into two subgroups. *T. dionisii* sequences exhibited two different profiles. According to the phylogenetic analysis, the sequences obtained from the molecular targets for the M1011 samples showed greater similarity with *T. dionisii* sequences obtained from insectivorous (*Myotis nigricans*) and frugivorous (*Sturnira lilium*) bats. With regard to the M1014 and M1015 isolates, the sequences obtained in this study showed greater similarity with *T. dionisii* sequences obtained from hematophagous (*D. rotundus*) and frugivorous (*Carollia perspicillata* and *Lophostoma brasiliense*) bats. It was not possible to determine any type of association on subgroups of *T. dionisii* and feeding habits from the information obtained from phylogenetic analysis. Hamilton et al. [28] demonstrated in a previous study which used phylogenetic analysis using 18S rRNA and *gGAPDH* sequences, clustered *T. dionisii* group A contained sequences from Europe only, and group B contained sequences from Europe and South America.

Phylogenetic analysis also confirmed that *T. dionisii* sequences from isolates obtained from hemathophagous and insectivorous bats were more similar to *T. c. cruzi* and *T. cruzi marinkellei* and closely related to *T. erneyi,* all species of the *Schizotrypanum* subgenus (Figure 2 and Supplementary Figure S2). Hamilton et al. [37] evaluated the relationships among *T. c. cruzi*, *T. cruzi marinkellei*, *T. dionisii*, and other species that comprise a single clade (clade *T. cruzi*), providing an interesting link between bats and *T. cruzi* evolution [26,38].

By multilocus enzyme electrophoresis (MLEE), random amplified polymorphic DNA (RAPD), and molecular sequencing, we found that the *T. dionisii* samples showed two different profiles, with samples M1014 and M1015 exhibiting the same profile and sample M1011 presenting a completely different one. Cavazzana et al. [4] demonstrated that there is only a 0.47% divergence in V7V8 of 18S DNA sequences from Brazilian *T. dionisii* isolates, and 2% divergence with *T. dionisii* from Europe [4].

Morphological analysis using light microscopy showed differences among the M1011, M1014, and M1015 samples, which were later confirmed with morphometric analysis. Another observed difference was that the isolates M1014 and M1015, naturally contained a higher percentage of trypomastigote forms compared to that of the isolate M1011. A blood smear test was also performed for the three samples, however, it was negative for all of them. Thus, it was not possible to compare the morphology of the parasites in axenic culture with parasites in blood smear.

Cimicids, the only recognized vectors of *T. dionisii,* have not been described in the state of Rio de Janeiro, however, we cannot confirm its absence. It is worth mentioning that *T. dionisii* has already been found in species of triatomines that could play a role in the transmission of this parasite, mainly through the oral route [17]. In this study, despite the three bats being from the same locality but different colonies, they could have been exposed to different vectors. In addition, numerous other vectors are involved in the transmission of different *Trypanosoma* species*,* including fleas [39], flies [40], sandflies [41], and triatomines [42]. It is not yet known which vectors are associated with bats and possibly act as disseminators of trypanosomatids that infect these animals. Both sandflies [43] and triatomines [44] are parasited by trypanosomatids that use bats as a host. Cave-type triatomines (*Cavernicola pilosa* and *Cavernicola lenti*) are described as possible vectors of some *Schizotrypanum* species, such as *T. cruzi marinkellei* [45]. An alternative possibility of direct transmission of *T. dionisii* among bats in this region should be considered due to their habit of aggregating into colonies [16]. The isolate M1011 was isolated from the insectivorous bat *L. aurita*, described as having cave-dwelling and colony-forming habits [46]. In a captive study, Thomas et al. [47] demonstrated that bats can be infected by different species of trypanosomes orally via the ingestion of triatomines as well as through contamination when exposed to the feces and urine of these insects or through bites. 

Due to little information available regarding the host specificity of most trypanosomes, the diversity of trypanosomes may have been considerably over- or underestimated with respect to their choice of host [28]. *T. dionisii*, previously believed to infect only bats and cimids, has been described in other mammalian hosts and vectors [17,18]. Therefore, a restricted association with its hosts cannot be guaranteed for any species of *Trypanosoma.* This leads us to question whether heterogeneity among isolates within the same species has to do with the adaptation to different hosts and if it is of medical importance to humans. To understand the true variability between *T. dionisii* isolates, studies integrating morphology, biology and molecular biology must be performed with isolates from different bat species and different locations.

## 4. Materials and Methods

### 4.1. Isolation and Light Microscopy of Samples

Samples were isolated from hemocultures from two hematophagous bats, *D. rotundus* (M1014 and M1015), and one insectivorous bat, *L. aurita* (M1011), which were caught in the municipal district of Miracema in Rio de Janeiro in 2007. All bat captures and blood collections were performed in association with the Bat Maintenance Program conducted by researchers from the Rio de Janeiro State Agricultural Research Corporation (PESAGRO-RIO). The bats were captured in wild and peri-urban areas at pre-established locations, such as near corrals, water shackles, basements, or natural bat shelters, employing mist-nets that were equipped at dusk. The captured bats were carefully removed from the nets and classified according to family, gender, and clinical appearance. For blood collection, the animals were anesthetized with ketamine at a concentration calculated according to body mass, and up to 1 mL of blood was collected via cardiac puncture. Afterward, the animals were identified with a collar and released at the same capture site [32].

The flagellated protozoa were isolated and maintained in biphasic medium NNN (Novy, McNeal, Nicolle) with Schneider and supplemented with 10% fetal bovine serum at 26–28 °C. The cultures were monitored weekly for 30 days. In all experiments, the three isolates were in the same passage in the axenic culture medium described above.

### 4.2. Parasite Morphology and Induction of Metacyclogenesis

Light microscopy was performed on Giemsa-stained smears using M1011, M1014, and M1015 parasites from culture with NNN and Schneider medium supplemented with 10% fetal bovine serum by three, 10, and 17 days. Additionally, epimastigote forms of the M1011 isolate were transferred at 1 × 10^6^ parasites/mL into Roswell Park Memorial Institute (RPMI) medium with 5% fetal calf serum at pH 8.0 for differentiation and acquisition of trypomastigote forms, following a protocol described by Koerich et al. [48]. The percentage of trypomastigote forms was determined by counting 100 randomly selected forms on the slides. Analyses were carried out under optical microscopy at 1000× magnification by examining the characteristics of the culture forms. All the experiment was performed in triplicate.

Measurements of the parasites (µm) were taken for both the total length of the cell body and the free flagellum of 20 randomly selected parasites. Photomicrographs were obtained using Motic Image Plus 2.0 software with the optical microscope Nikon Eclipse E200. Statistical analysis was performed to compare the values of body and flagellum measurements of M1011 and M1014 samples using a one-way ANOVA test. The null hypothesis for the test was that the two means were equal, and a comparison of means whose alpha decision level was equal to or less than 0.05 was used.

### 4.3. Multilocus Enzyme Electrophoresis (MLEE)

The isoenzyme electrophoresis technique was used in accordance with the protocols described by Cupolillo et al. [49]. Nine enzymatic systems were analyzed; nucleotidase (NH), phosphoglucomutase (PGM), mannose phosphate dehydrogenase (MPI), 6-phosphogluconate dehydrogenase (6PGDH), malic enzyme (ME), glucose phosphate isomerase (GPI), glucose-6-phosphate dehydrogenase (G6PDH), malate dehydrogenase (MDH), and isocitrate dehydrogenase (IDH). The electrophoretic mobility of the isolates was compared to the reference samples; *T. c. cruzi* DTUs TcII and TcVI (Y and CL strains, respectively), *Trypanosoma rangeli* (Choachi strain), and *Trypanosoma desterrensis* [50].

### 4.4. Polymerase Chain Reaction (PCR) Assays 

PCR assays were performed using DNA extracted from cultured trypanosomes. Approximately 20 mL of culture were centrifuged and washed twice in sterile phosphate buffer saline (1 M; pH 7.2) to pellet the cells, and DNA was extracted using DNAzol kit (Invitrogen, USA) according to the manufacturer’s instructions.

PCR amplification of V7V8 18S rDNA was performed to amplify an approximately a 900 bp fragment under conditions described by Marcili et al. [34]. The *gGAPDH* gene from the isolates was amplified using primers G1/G2 (900 bp), as described by Hamilton et al. [35]. The primers used to amplify *Cytb* gene region encoded in the maxicircle DNA of the mitochondrial genome were P18/P20 (500 bp) [36]. In all assays, water was used as negative control and DNA from *T. c. cruzi* DTU TcII (Y strain) as a positive control. The PCR products were purified using the QIAquick Purification Kit (Qiagen, Manchester, UK) to prepare them for sequencing.

### 4.5. Sequencing Analysis

All nucleotide sequences were determined using an automatic sequencer (3730 DNA Analyzer, Applied Biosystems) and analyzed using the Basic Local Alignment Search Tool (BLAST) program (http://blast.ncbi.nlm.nih.gov/Blast.cg). All nucleotide sequences obtained for each molecular target (V7V8 18S rDNA, *gGAPDH*, and *Cytb*) were aligned and compared using the Molecular Evolutionary Genetics Analysis (MEGA) program version 7.0.26 [51] with bat trypanosome species and other trypanosomatids available from GenBank (https://www.ncbi.nlm.nih.gov/pmc/articles/PMC540017/). 

The concatenated alignment V7V8 18S rDNA and gGAPDH results were analyzed by neighbor-joining (NJ), maximum likelihood (ML), and Bayesian (B) methods using Kimura 2-parameter model with gamma distributed rate variation. To evaluate the robustness of the nodes in the resulting phylogenetic tree for NJ and ML methods, 1000 bootstrap replications were performed using MEGA version 7.0.26 [52]. The models of sequence evolution and their parameters were calculated using the jModelTest in MEGA version 7.0.26 [52]. Bayesian inference (BI) was run in MrBayes (version 3.1.1) [53] with a Kimura 2-parameter model with gamma distributed rate variation. The runs converged after 1,000,000 generations and discarding the first 25% of the trees as burn-in. Phylogenetic tree inferred by Maximum likelihood with the Tamura-Nei model of partial sequences of *Cytb* genes of *T dionisii* from this study and reference sequences deposited in GenBank were aligned with the MEGA program version 7.0.26 [53]. Sequences of bat trypanosomes from this study, the species included in the phylogenetic trees and their respective host, geographical origin, and GenBank accession numbers are shown in Table 1.

### 4.6. Random Amplified Polymorphic DNA (RAPD)

The genetic variability among isolates was assessed by random DNA amplification using four arbitrary sequence primers with 10 nucleotides (Pharmacia Biothec Ready-To-Go^TM^RAPD Analysis Kit), according to the manufacturer’s recommendations. The amplified products were analyzed on an agarose gel (2%), stained with ethidium bromide, and visualized under ultraviolet light. The RAPD profiles were analyzed using the Jaccard similarity coefficient to determine the proportion of similar pieces among all isolates. The matrix was transformed into a dendrogram by an unweighted method of grouping in pairs using the arithmetic mean (unweighted pair group method using arithmetic averages (UPGMA)) [54]. Numerical analysis was performed using the NTSYS-pc software program (Version1.70, Exeter software).

## Figures and Tables

**Table 1:** GenBank accession numbers of sequences of different genes employed for analyses from different *Trypanosoma* spp., hosts and geographic origin. * samples from this study. BR—Brazil, BO—Bolivia, MZ—Mozambique, 18S rDNA—small subunit rDNA, *gGAPDH*—Glyceraldehyde-3-phosphate dehydrogenase, *Cytb*—cytochrome *b*.

*Trypanosoma* spp. Isolates	Host Species	Geographic Origin	GenBank Accession Number		
			18S rDNA	*gGAPDH*	*Cytb*
***T. dionisii***					
M1011 *	*Lonchorhina aurita*	Rio de Janeiro/BR	MH047820	MN233645	MH047823
M1014 *	*Desmodus rotundus*	Rio de Janeiro/BR	MH047821	MN233646	MH047824
M1015 *	*Desmodus rotundus*	Rio de Janeiro/BR	MH047822	MN233647	MH047825
CBT 116	*Myotis nigricans*	Espírito Santo/BR	KF557751	KF557742	-
CBT 69	*Desmodus rotundus*	Espírito Santo/BR	KF557750	KF557741	-
CBT 66	*Carollia perspicillata*	Espírito Santo/BR	KF557749	KF557740	-
CBT 65	*Carollia perspicillata*	Espírito Santo/BR	KF557748	KF557739	-
CBT 64	*Carollia perspicillata*	Espírito Santo/BR	KF557747	KF557738	-
CBT 63	*Carollia perspicillata*	Espírito Santo/BR	KF557746	KF557737	-
CBT 59	*Lophostoma braziliense*	Espírito Santo/BR	KF557745	KF557736	-
CBT 58	*Sturnira lillium*	Espírito Santo/BR	KF557744	KF557735	-
P3	*Pipistrellus pipistrellus*	England	AJ009151	AJ620271	-
X842	*Nyctalus noctula*	England	FN599058	FN599055	-
MPM < JPN > :21385	*Miniopterus fuliginosus*	Japan	LC326397	LC326399	-
SD044	*Eptesicus serotinus*	China	MH393943	MH393931	-
TryCC 1087	*Sturnira lillium*	Goiás/BR	-	-	FJ900253
TryCC 1314	*Sturnira lillium*	Paraná/BR	-	-	FJ900254
TryCC 1059	*Eptesicus brasiliensis*	Tocantins/BR	-	-	FJ900252
TryCC 211	*Eptesicus brasiliensis*	São Paulo/BR	-	-	FJ900249
TryCC 454	*Desmodus rotundus*	Mato Grosso do Sul/BR	-	-	FJ900250
TryCC 495	*Carollia perspicillata*	Roraima/BR	-	-	FJ900251
TryCC 1110	*Carollia perspicillata*	São Paulo/BR	-	-	FJ002263
272	*Carollia perspicillata*	Guacharos/BO	-	-	JN651290
274	*Carollia perspicillata*	Guacharos/BO	-	-	JN651291
***T. erneyi***					
TCC 1293	*Tadarida* sp.	Chupanga/MZ	JN040987	JN040964	JN040956
TCC 1936	*Mops condylurus*	Chupanga/MZ	-	-	JN040960
***T. c. marinkellei***					
TCC 344	*Carollia perspicillata*	Rondonia/BR	FJ001664	GQ140360	KT327330
TCC 501	*Carollia perspicillata*	Rondonia/BR	FJ001665	GQ140361	JN543702
***T. c. cruzi***					
TryCC 793	*Myotis levis*	São Paulo/BR	FJ900241	GQ140358	-
TryCC 507	*Carollia perspicillata*	Amazonas/BR	FJ900240	GQ140352	FJ002256
TCC1994	*Myotis levis*	São Paulo/BR	-	-	KT327329
***T. rangeli***					
TCC 643	*Platyrrhinus lineatus*	Mato Grosso do Sul/BR	FJ900242	GQ140364	-
TryCC 643	*Platyrrhinus lineatus*	Mato Grosso do Sul/BR	-	-	JN040963
***T. vespertilionis***					
P14	*Pipistrellus pipistrellus*	England	AJ009166	AJ620283	-

## Supplementary Materials

The following are available online at https://www.mdpi.com/2076-0817/9/9/736/s1, Figure S1: Schematic representation of the isoenzyme patterns for glucose phosphate isomerase (GPI), malate dehydrogenase (MDH), isocitrate dehydrogenase (IDH), malic enzyme (ME). A—*Trypanosoma rangeli* (Choachi), B and C—*Trypanosoma c. cruzi* (Y, CL Brener), D—*Trypanosoma desterrensis*, E, F and G—*Trypanosoma dionisii* (M1011, M1014, M1015). Figure S2: Phylogenetic analyses demonstrating the clear separation of *Trypanosoma dionisii* isolates into two groups. Phylogenetic tree inferred by Maximum likelihood with the Tamura–Nei model of partial sequences of cytochrome *b* genes of *T dionisii* from this study and reference sequences deposited in GenBank were aligned with the Molecular Evolutionary Genetics Analysis (MEGA) program version 7.0.26. Outgroup: *Trypanosoma rangeli.* Bootstrap test results (1000 replicates) are shown next to the branches GenBank accession numbers are shown in Table 1. Figure S3: Unweighted pair group method using arithmetic averages (UPGMA) dendrogram built with the simple matching coefficient of similarity based on the genetic profiles obtained from Random Amplified Polymorphic (RAPD) among isolates. *T. dionisii* M1011 was clustered separated M1014 and M1015 samples. *T. c. cruzi* Y, *T. cruzi* ClBrenner, *T. rangeli* Choachi and *T. desterrensis* were used as reference and were clustered totally separated of the *T. dionisii* samples. Table S1: The sequence analysis of DNA products obtained with the three molecular targets in comparison with DNA sequences of *Trypanosoma* spp. deposited in GenBank confirmed the identification of the three isolates as *Trypanosoma dionisii*. Graphic S1: Body and flagellum measurements of parasites used in morphometric analysis. A. M1011, B. M1014.
