# Supplementary material for: Biological and Genetic Heterogeneity in Trypanosoma dionisii Isolates from Hematophagous and Insectivorous Bats
Source: Pathogens. 2020 Sep 7;9(9):736. doi: 10.3390/pathogens9090736 (PMC7558101; doi:10.3390/pathogens9090736)

Supplementary Table S1: The sequence analysis of DNA products obtained with the three molecular targets in comparison with DNA sequences of *Trypanosoma* spp. deposited in GenBank confirmed the identification of the three isolates as *Trypanosoma dionisii.*

Molecular target	Samples	Identity (%)	Query cover (%)	Reference sequence (*T. dionisii*)
18S rDNA	M1011	100	100	KF557744
	M1014	99.84	100	KF557750
	M1015	99.84	100	KF557750
gGAPDH	M1011	99.29	100	GQ140362
	M1014	100	100	MG471432
	M1015	100	100	MG471432
Cytb	M1011	100	100	KF557750
	M1014	100	95	MG471432
	M1015	100	94	FJ900251

For all sequences e-value = 0.0

Supplementary Figure S1: Schematic representation of the isoenzyme patterns for glucose phosphate isomerase (GPI), malate dehydrogenase (MDH), isocitrate dehydrogenase (IDH), malic enzyme (ME). A – *Trypanosoma rangeli* (Choachi), B-C– *Trypanosoma c. cruzi* (Y, CL Brener), D – *Trypanosoma desterrensis*, E, F and G – *Trypanosoma dionisii* (M1011, M1014, M1015).

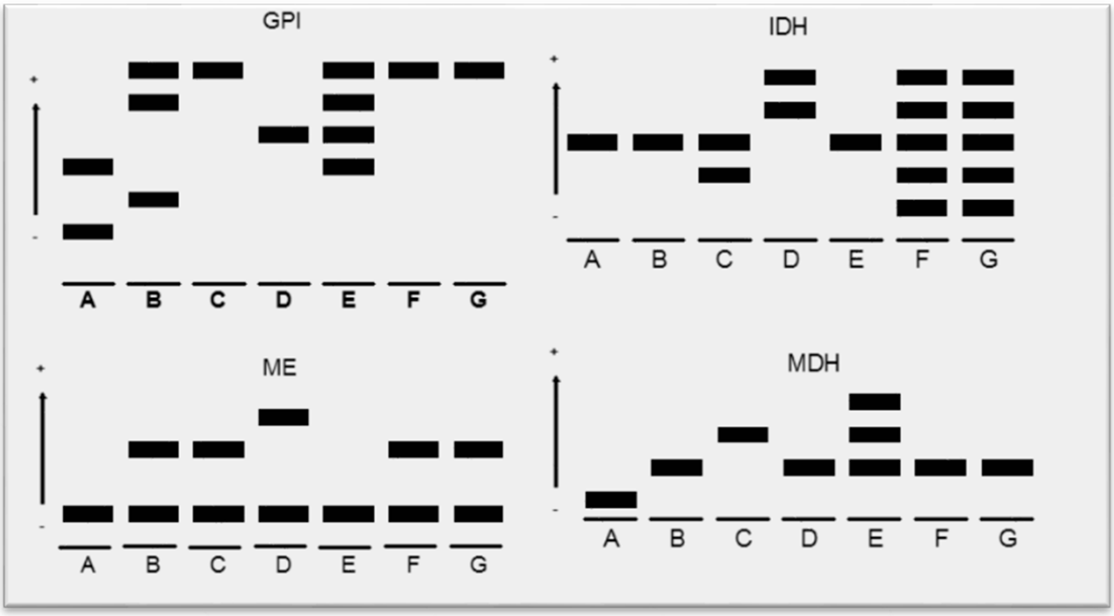

Supplementary Figure S2: Phylogenetic analyses demonstrating the clear separation of *Trypanosoma dionisii* isolates into two groups. Phylogenetic tree inferred by Maximum likelihood with the Tamura–Nei model of partial sequences of cytochrome b genes of *T dionisii* from this study and reference sequences deposited in GenBank were aligned with the Molecular Evolutionary Genetics Analysis (MEGA) program version 7.0.26. Outgroup: *Trypanosoma rangeli.* Bootstrap test results (1000 replicates) are shown next to the branches GenBank accession numbers are shown in Table 1.

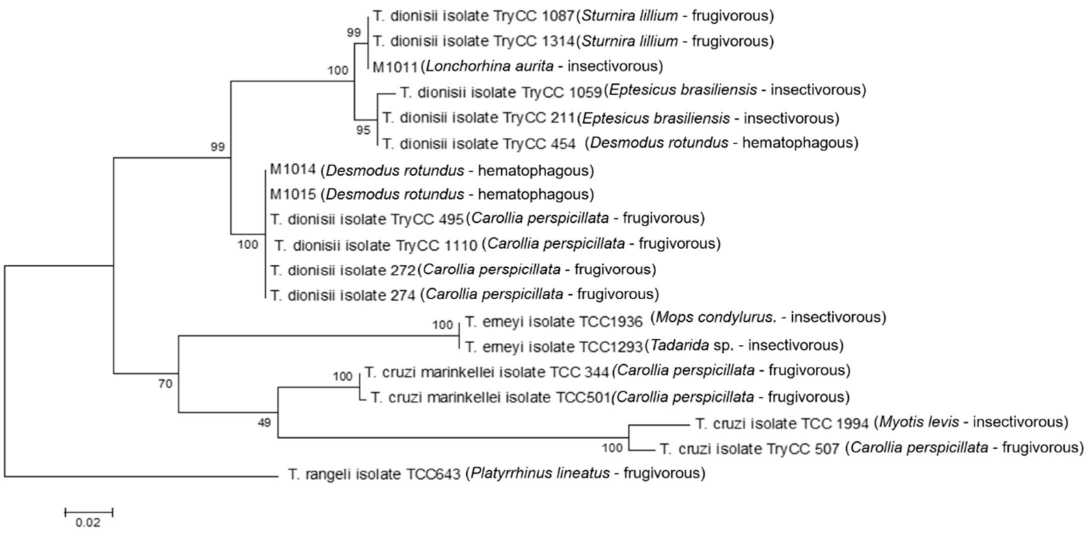

Supplementary Figure S3: Unweighted pair group method using arithmetic averages (UPGMA) dendrogram built with the simple matching coefficient of similarity based on the genetic profiles obtained from Random Amplified Polymorphic (RAPD) among isolates. *T. dionisii* M1011 was clustered separated M1014 and M1015 samples. *T. c. cruzi* Y, *T. cruzi* ClBrenner, *T. rangeli* Choachi and *T. desterrensis* were used as reference and were clustered totally separated of the *T. dionisii* samples.

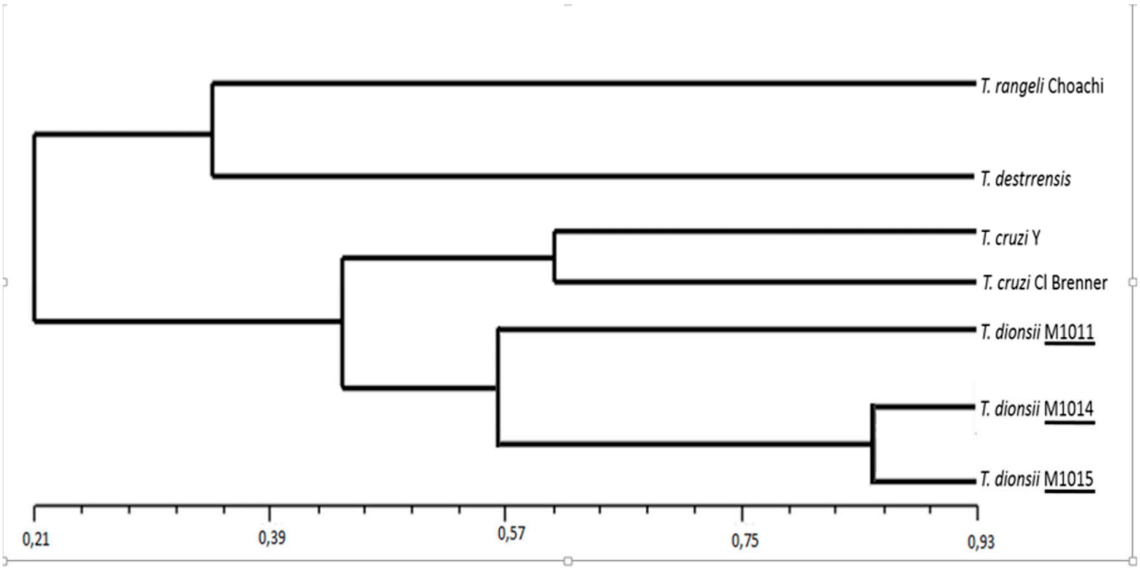

Supplementary Graphic S1: Body and flagellum measurements of parasites used in morphometric analysis. A. M1011, B. M1014.

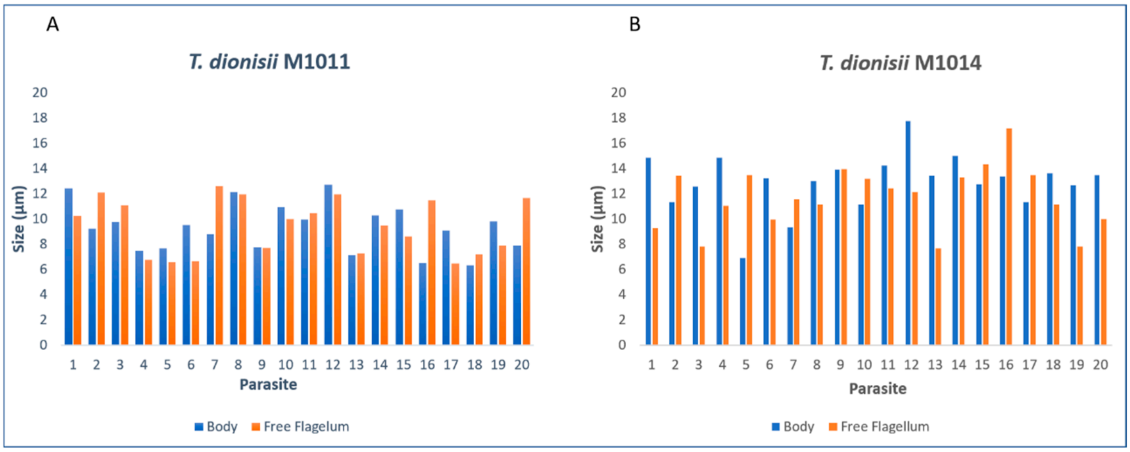

Supplement: Supplementary file 1 [file pathogens-09-00736-s001.pdf]
